# Changes in the Mechanical Properties of Nickel–Titanium Orthodontic Archwires After Clinical Use with Conventional and Self-Ligating Brackets

**DOI:** 10.3390/dj14060351

**Published:** 2026-06-08

**Authors:** Guillem Ruiz, Javier Moyano, Inés Alcaraz, Núria Clusellas, Núria Molina, Javier Gil, Montserrat Artés, Andreu Puigdollers

**Affiliations:** 1 Department of Orthodontics, School of Dentistry, Universitat Internacional de Catalunya, Sant Cugat del Vallés, 08195 Barcelona, Spain; guillemruiz@uic.es (G.R.); inesalcaraz@uic.es (I.A.); nclusellas@uic.es (N.C.); nuriamolina@uic.es (N.M.); martes@uic.es (M.A.); apuigdollersp@gmail.com (A.P.); 2 Biomimetics Oral Biomaterials and Interfaces (BOBI), Department Ciencia e Ingeniería de Materiales, Escola d’Enginyeria Barcelona Est, Universitat Politècnica de Catalunya, c/Eduard Maristany 16, 08029 Barcelona, Spain

**Keywords:** NiTi, superelasticity, self-ligating brackets, mechanical properties

## Abstract

**Background/Objectives**: Changes in the mechanical behavior of orthodontic archwires during clinical use are not fully understood, particularly when different bracket systems are employed. Self-ligating (SL) brackets have gained considerable popularity in orthodontic practice in recent years, largely due to claims of improved treatment efficiency and biomechanical performance. Nevertheless, current evidence has not consistently demonstrated statistically significant differences between conventional ligation (CL) brackets and SL systems. The aim of this study was to evaluate changes in the mechanical properties and degradation over time of nickel-titanium (NiTi) archwires after clinical use in orthodontic treatments performed with CL and SL brackets. **Methods**: A comparative study was conducted using archwires retrieved from orthodontic patients. Round 0.014-inch NiTi wires (GC Orthodontics America Inc., IL, USA) were analyzed. The archwires were used in 60 patients treated with either CL or SL appliances and evaluated at four time points: before clinical use (T0), and after 1 month (T1), 2 months (T2), and 3 months (T3) of intraoral service. Mechanical testing was performed according to ISO 15841:2014 + Amd. 1:2020 using a three-point bending test with a universal testing machine (Z005 Test Control II Universal Testing Machine, Zwick Roell, Kennesaw, GA, USA). The variables analyzed included the mean force delivered by the archwires at deflections of 3 mm (F3), 2 mm (F2), 1 mm (F1), and 0.5 mm (F0.5), as well as the slope of the superelastic plateau at 2 mm, 1 mm, and 0.5 mm. The static and dynamic friction coefficients, as well as the friction forces associated with the wires and the two types of brackets, were determined using a modified MTS-Bionix servo-hydraulic testing machine. The tests were conducted at 37 °C in a saline environment. **Results**: Both groups showed changes in the superelastic behavior of NiTi archwires. Alterations increased with longer intraoral exposure. In the SL group, significant modifications were already observed after one month of clinical use, with a reduction in the force delivered and a loss of superelastic characteristics. These changes remained relatively stable thereafter, with no statistically significant differences during the following months. In contrast, the CL group showed a progressive reduction in force delivery and superelasticity over time. This is due to the difference in friction between the wire and the CL bracket compared to the SL bracket, which results in greater force transfer for tooth movement. **Conclusions**: Overall, differences in the mechanical behavior of archwires between CL and SL systems were observed during the initial stages of clinical use. However, these differences diminished over time, and no significant differences were detected after three months. Considering the progressive degradation of mechanical properties, the reuse of archwires that have remained intraorally for more than three months may not be advisable.

## 1. Introduction

Advances in orthodontic mechanotherapy have been largely driven by the development of new archwire materials [1,2,3]. Currently, clinicians can select from a wide range of wires designed to meet specific clinical requirements. Appropriate selection of wire dimensions and alloy composition plays a key role in achieving efficient, predictable, and biologically acceptable treatment outcomes [1,4,5,6]. A thorough understanding of the mechanical behavior of orthodontic wires enables clinicians to optimize tooth movement while minimizing potential damage to the dentition and periodontal tissues.

Light and continuous forces are generally considered the most favorable in orthodontic therapy, as they are associated with reduced patient discomfort and a lower risk of tissue hyalinization and root resorption [3,7,8,9]. Among orthodontic materials, nickel–titanium (NiTi) alloys have become particularly important due to their unique mechanical characteristics.

Superelastic NiTi wires exhibit constant, low stresses that promote tissue movement [9,10]. These stresses can be modified by chemical composition, crystal size, or accumulated internal stresses. In general, the wires exhibit the austenitic or beta phase, which is responsible for the superelastic behavior, although some wires exhibit the so-called R phase, which is considered a premartensitic phase that also exhibits good elastic behavior. The martensitic phase is the phase that exhibits plastic behavior, characterized by permanent deformation; when heated to the austenite range, it reverts to the austenitic phase and regains the original shape with which it was manufactured [11,12,13]. The reversible transformation between austenite and martensite is responsible for the two most distinctive properties of NiTi archwires: superelasticity and shape memory effect [13,14,15]. These characteristics represent major advantages over conventional metallic alloys [15]. Importantly, phase transformation in NiTi alloys occurs at relatively low transition temperatures compared to stainless steel and other metallic materials. This transformation can be triggered either by temperature changes or by the application of mechanical stress within a specific temperature range [7,13,16].

Although extensive research has investigated the mechanical properties and phase transformation behavior of NiTi wires under laboratory conditions [7,13,14,17,18,19,20], limited evidence exists regarding the influence of the intraoral environment on their mechanical performance. Eliades et al. [21] suggested that the scarcity of in vivo evidence may be related to the inherent limitations of in vitro models, which are unable to fully reproduce the complex biological, chemical, and mechanical conditions present in the oral cavity.

In recent years, self-ligating (SL) brackets have become increasingly popular in orthodontic practice, supported by claims of improved efficiency and clinical performance. Manufacturers and distributors have attributed multiple advantages to SL systems, including reduced friction, faster archwire changes, improved engagement, enhanced patient comfort and hygiene, fewer emergency visits, reduced root resorption, decreased need for extractions due to expansion, shorter treatment duration, fewer appointments, and overall improved clinical efficiency [22]. However, evidence from independent studies has primarily confirmed reduced ligation time as the main advantage of SL brackets when compared with conventional ligation (CL) systems, while most other proposed benefits have not demonstrated statistically significant differences [22,23,24,25].

Therefore, the objective of this study was to evaluate and compare, under real clinical conditions, the changes in force delivery superelastic properties and degradation of NiTi archwires when used with conventional ligation (CL) and self-ligating (SL) bracket systems after three months of clinical use, that is, the time that an orthodontic wire usually stays in the mouth during an orthodontic treatment.

The null hypothesis of this study states that there are no statistically significant differences in the superelastic properties of NiTi orthodontic archwires after three months of clinical use between conventional ligation (CL) and self-ligating (SL) bracket systems.

## 2. Materials and Methods

The sample consisted of superelastic nickel–titanium (NiTi) orthodontic archwires with a round cross-section of 0.014 inch (0.356 mm) obtained from a single manufacturer (GC Orthodontics America Inc., Alsip, IL, USA). Archwires were evaluated in 60 consecutive patients undergoing orthodontic treatment at the University Dental Clinic (CUO, Universitat Internacional de Catalunya, Barcelona, Spain). The archwire evaluated in each case corresponded to the first archwire placed at the beginning of orthodontic treatment. Patients were included if they were healthy young adults aged 18–30 years, presented with low-to-moderate lower arch crowding, good oral hygiene, a full permanent dentition, and a mesofacial growth pattern; full ligation of the wire was required at every visit. Patients were excluded if they presented any systemic disease, bruxism, periodontal disease, previous orthodontic treatment, poor oral hygiene, missing teeth, or incomplete wire ligation at any visit.

The study protocol was approved by the Clinical Research Ethics Committee of the Universitat Internacional de Catalunya (ORT-ELM-2019-01). Measurements were performed at four time points: before clinical use (T0), after one month of intraoral use (T1), after two months (T2), and after three months (T3).

Thus, the total sample consisted of 10 archwires used as a control group (T0) and an additional 10 archwires evaluated at each intraoral aging period (T1, T2, and T3) for both bracket systems: conventional ligation (CL) and self-ligating (SL). Table 1 shows the sample distribution.

In the CL group, stainless steel brackets with a 0.022-inch slot and MBT prescription were used (Victory Series MBT 0.022, 3M, Saint Paul, MN, USA). In order to minimize the potential confounding effect of elastomeric ligature degradation [26], patients in this group were recalled on a monthly basis, and elastomeric ligatures were replaced at each visit for those wearing the archwire for more than one month. In the SL group, self-ligating brackets with a 0.022-inch slot and CCO prescription were used (Experience Metal Self-Ligating CCO 0.022, GC Orthodontics America Inc., Alsip, IL, USA).

Sample size calculation indicated that, assuming a significance level (α) of 0.05 and a statistical power of 80% (β = 0.20) in a two-sided test, a minimum of 10 subjects per group would be required to detect a difference of at least 0.05 N. The standard deviation was assumed to be 0.25 based on previous studies [27].

**Figure 1:**
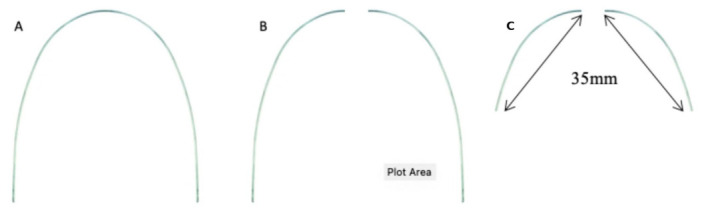
(**A**) Archwire tested. (**B**) Segmentation: 2 samples of 1 archwire. (**C**) Parts of the archwire were studied.

All archwires were randomly assigned to patients. Mechanical properties were evaluated before clinical use (T0) and after one (T1), two (T2), and three months (T3) of intraoral service. After retrieval, 10 archwires at each period of time (T1, T2, T3) were sectioned into two halves, providing two specimens per archwire. This resulted in a total of 20 testing (n = 20) points for each experimental group (Figure 1).

**Figure 2:**
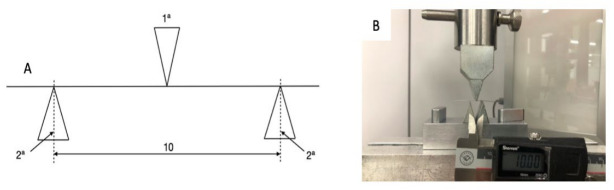
(**A**) Sketch from ISO 15841:2014 + Amd. 1:2020 1^a^ Central loading roller 2^a^ Support rollers [28]/(**B**) Picture taken during the test.

Analysis of the tensile properties of all the archwire samples at different times (T0, T1, T2 and T3) and different techniques (CL and SL) was carried out by a three-point bending test with a universal testing machine (Z005 Test Control II Universal Testing Machine; Zwick Roell, Kennesaw, GA, USA) (Figure 2).

The middle portion of the wire segment was deflected at a crosshead speed of 7.5 mm/min under the pressure from a metal penetrator point. Each sample was loaded until 3.1 mm of deflection of the middle portion of the wire. The degree of deflection was similar to the clinical situation of the leveling and aligning phase. The wire segments were unloaded at the same crosshead speed until the released force reached zero. Subsequently, the unloading curve and the superelasticity of each wire segment were evaluated using the following nine parameters:

- Force level delivered in N when the deflection is 3.0 mm, 2.0 mm, 1.0 mm, 0.5 mm (Fdef-3 mm, Fdef-2 mm, Fdef-1 mm, Fdef-0.5 mm, respectively).
- The deflection of the archwire at the end of the plateau in mm (Sp).
- Plateau slopes; between 0.5 mm and Sp (Slope-0.5 mm), between 1 mm and Sp (Slope-1 mm), and between 2 mm and Sp (Slope-2 mm) of deflection expressed in N/mm.

**Figure 3:**
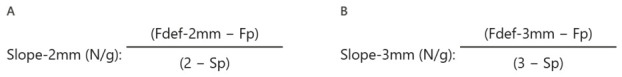
Formula to obtain Plateau Slopes from 2 mm (**A**) and 3 mm (**B**) of deactivation, respectively.

Plateau slope is a measurement of the degree of plateau flatness; therefore, the closer the slope is to zero, the more constant the force is. The formulas are expressed in Figure 3A,B.

**Figure 4:**
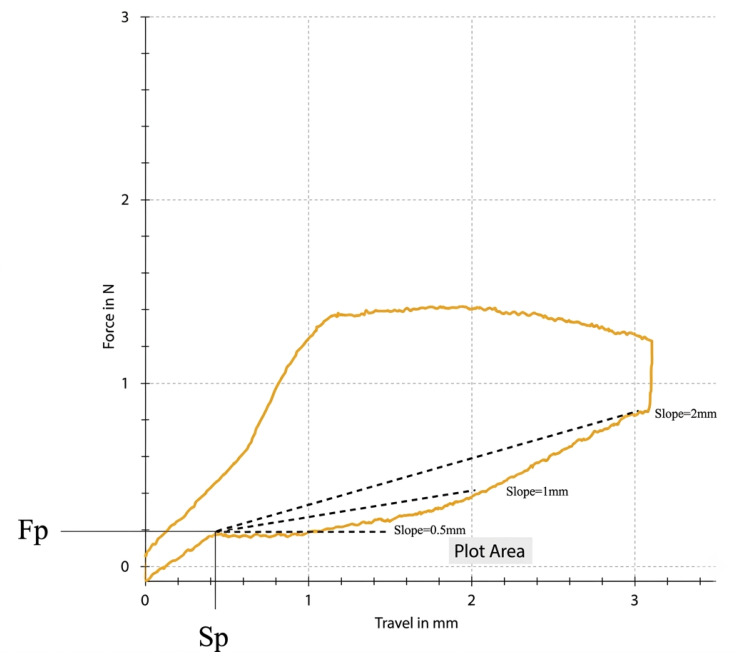
Parameters measured were represented on a graph obtained. Deflection (Sp) and force delivered (Fp) at the end of the plateau. Plateau Slope from 2 mm (Slope-2 mm) and 3 mm (Slope-3 mm) of deflection.

A graph for each archwire studied was obtained, Figure 4. On the x-axis, it represented the deformation in millimeters (mm). On the abscissa it represented the force exerted in Newtons (N). The limit of the x-axis was placed at 3.5 mm, while the y-axis upper limit was placed at 4 N. These values were chosen because between these limits all loading and unloading curves were represented. Therefore, representing all graphs with the same limits, it is possible to visually compare different archwires. Furthermore, it allows overlapping graphs from one archwire to another.

The coefficients of friction (static and dynamic) were studied for 20 orthodontic wires with CL and SL brackets, 10 for each type of bracket. To determine the sample size, the following method was used: ‘Inference for Means: Comparing Two Independent Samples’ website developed by the Department of Statistics at the Univ. of British” Columbia “http://www.stat.ubc.ca/~rollin/stats/ssize/n2.html (accessed on 25 February 2025)”. A sample size of 7 was calculated for a desired power of 0.80 and a significance level of *p*-value < 0.05. In this work, we indeed tested 10 brackets for each type, which is a sample size larger than the minimum necessary for the desired power. The friction force values for each wire were determined in a bath of saline solution at 37 °C. The chemical composition is 0.9% NaCl by volume in water. Several studies have shown that the mechanical properties are not affected by whether artificial saliva or saline solution is used [29,30].

The determination of friction coefficients using a sliding test machine consists of evaluating the resistance to motion between two contacting surfaces. During the test, the machine applies a controlled normal load and generates relative motion between the specimens, one bracket at a time, while sensors measure the resulting friction force. All measurements were repeated with 10 different wires and brackets. Using these data, the friction coefficient is calculated as the ratio between the friction force and the normal force. This procedure allows the determination of both static and dynamic friction coefficients. In the system used, an oscillatory motion with a frequency of 10 Hz occurs between the wire and the bracket we wish to study, and a normal load of 10 N is applied. The force detected by the sensor during the initial movement corresponds to the static friction force, and once the system is in motion, it corresponds to the dynamic friction force. Knowing the normal load, the coefficients of friction can be calculated. These experiments were conducted with the test system immersed in saline solution at a constant temperature of 37 °C. It should be noted that in this test, the motion is linear, as specified by the international standard ISO 15841:2014/Amd 1:2020 [28].

**Figure 5:**
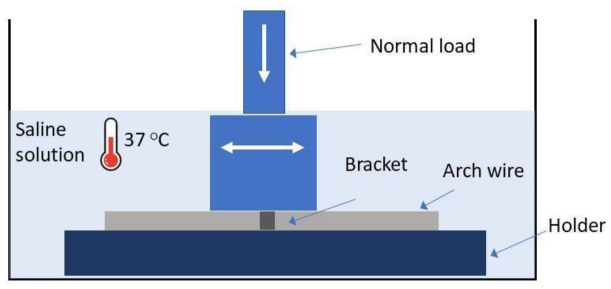
Scheme of the equipment to determine the static and dynamic coefficients for the system NiTi archwires and the CL and SL brackets used in this study.

Figure 5 shows a diagram of the testing machine used, based on the MTS Bionix (Minneapolis, MN, USA), employing a deformation rate of 0.1 mm/min to simulate in-service behavior as closely as possible.

Results were analyzed using Jamovi software (The jamovi project 2022. Jamovi Version 2.3, Computer Software www.jamovi.org). Firstly, the descriptive statistics were carried out with means and standard deviations for each measurement. Normality was tested using the Shapiro–Wilk test, and the One-way Analysis of Variance (ANOVA) was applied to compare mean differences among different periods of time. Additional mean values obtained in each technique were tested with a Student’s *t*-test. A Bonferroni correction was applied to control the familywise error rate. The corrected significance threshold was set at α = 0.0023 (0.05/21), and only comparisons yielding *p*-values below this threshold were considered statistically significant.

## 3. Results

### 3.1. Conventional Ligation Brackets (CL)

**Figure 6:**
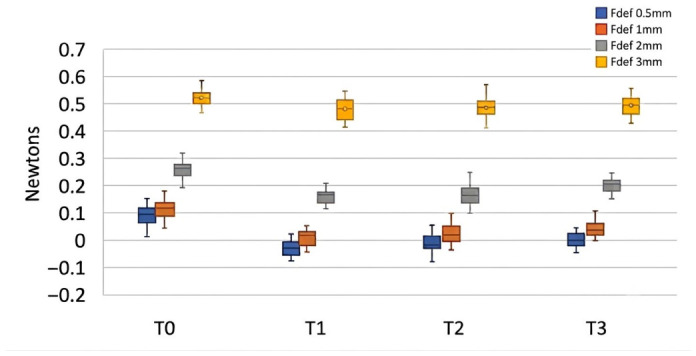
Boxplot diagram showing force delivered (N) in the CL group.

**Figure 7:**
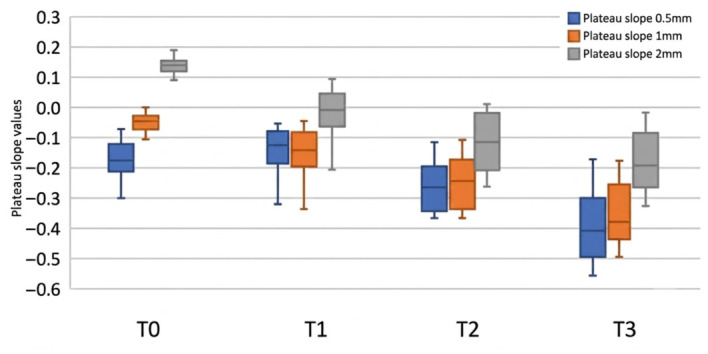
Boxplot diagram showing plateau slope in the CL group.

The average data for the force delivered (N) by the orthodontic archwires for the deflections of 3, 2, 1 and 0.5 mm and the plateau slopes for 0.5 mm, 1 mm and 2 mm are shown in Table 2 and Table 3 and Figure 6 and Figure 7. These data provide a comprehensive understanding of the amount of force generated by CL in a specific range of deflection and the superelastic properties of it after a 3-month period in the oral cavity.

### 3.2. Self-Ligating Brackets (SL)

**Figure 8:**
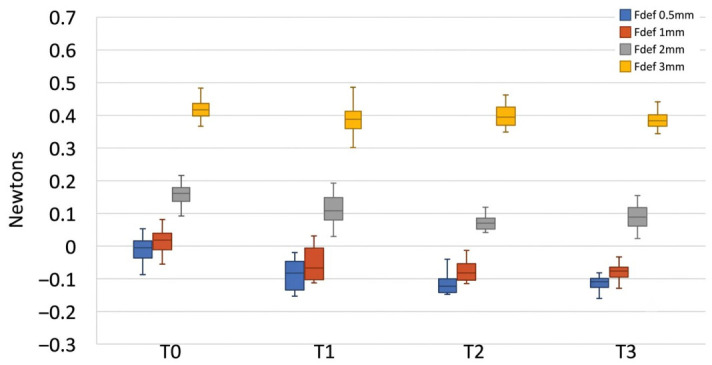
Boxplot diagram showing force delivered (N) in the SL group.

**Figure 9:**
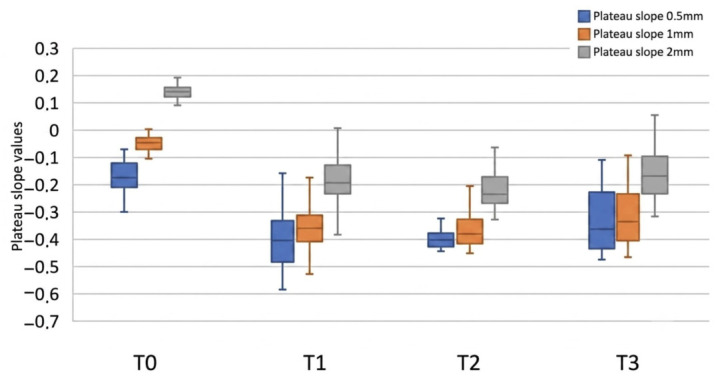
Boxplot diagram showing plateau slope in the SL group.

The mechanical behavior of archwires used with SL brackets is presented in Table 4 and Table 5 and Figure 8 and Figure 9, which display the mean forces (N) recorded at deflections of 3, 2, 1 and 0.5 mm, together with the plateau slopes at 0.5, 1 and 2 mm.

### 3.3. Conventional Ligation Brackets (CL Compared to Self-Ligating Brackets (SL)

**Figure 10:**
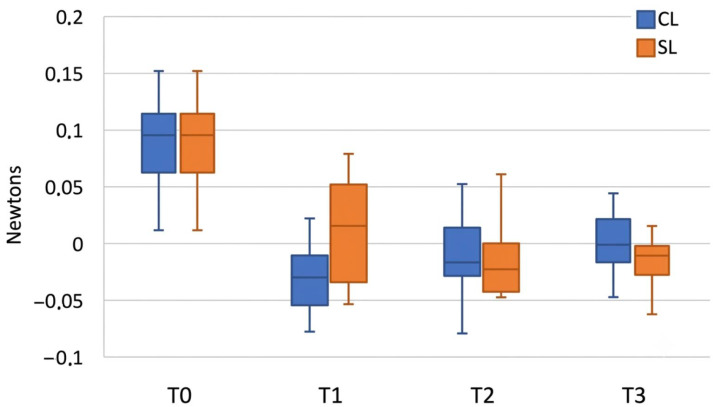
Boxplot diagram comparing Fdef 0.5 mm (N) between CL and SL.

**Figure 11:**
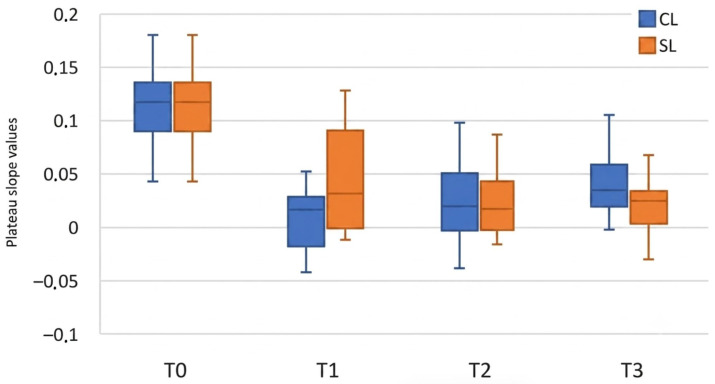
Boxplot diagram comparing Fdef 1 mm (N) between CL and SL.

**Figure 12:**
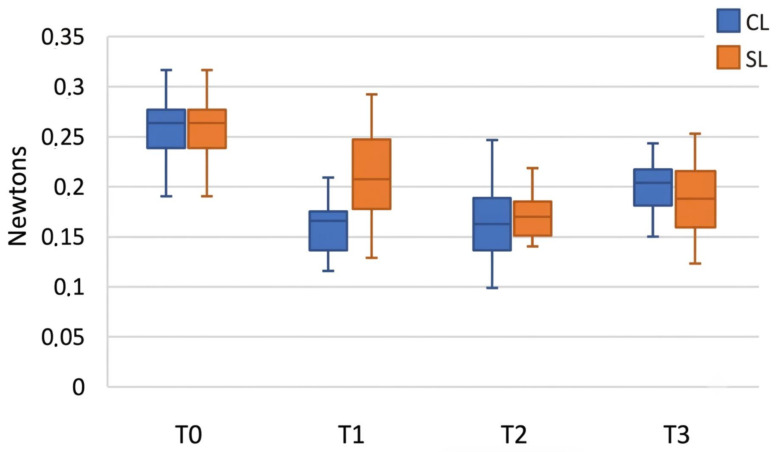
Boxplot diagram comparing Fdef 2 mm (N) between CL and SL.

**Figure 13:**
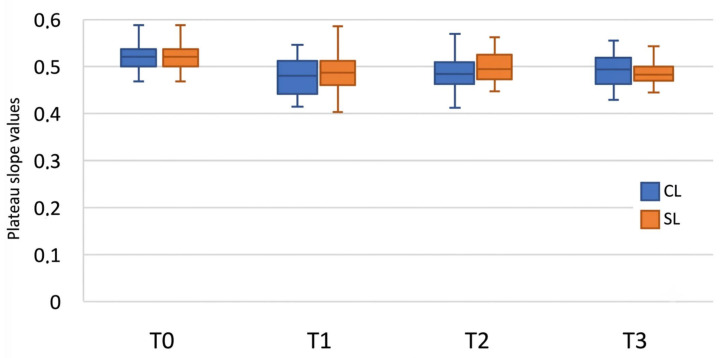
Boxplot diagram comparing Fdef 3 mm (N) between CL and SL.

**Figure 14:**
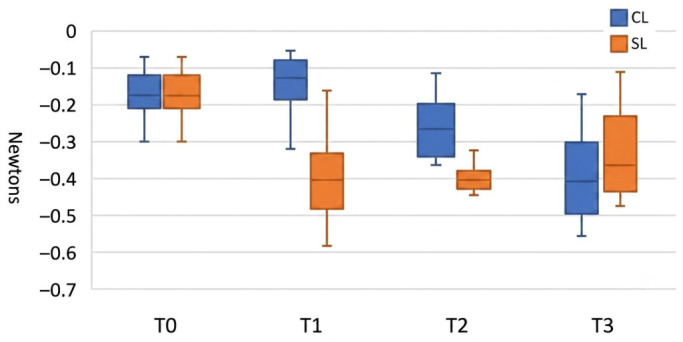
Boxplot diagram comparing plateau slope 0.5 mm between CL and SL.

**Figure 15:**
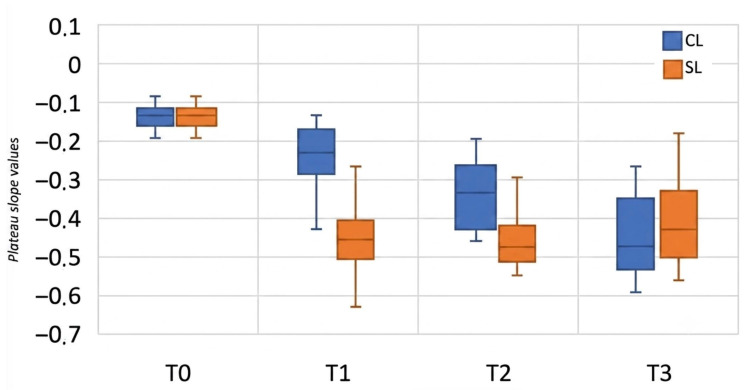
Boxplot diagram comparing plateau slope 1 mm between CL and SL.

**Figure 16:**
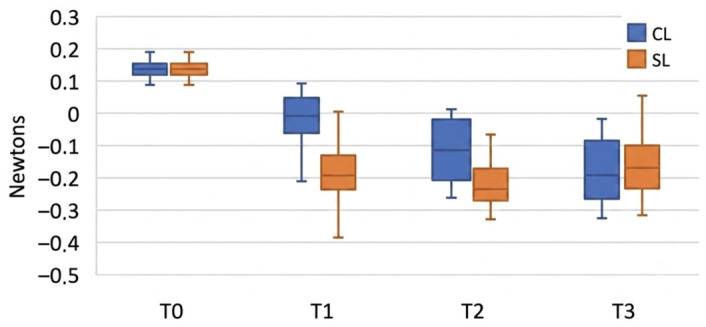
Boxplot diagram comparing plateau slope 2 mm between CL and SL.

Table 6 and Table 7 and Figure 10, Figure 11, Figure 12, Figure 13, Figure 14, Figure 15 and Figure 16 show changes in the properties between CL and SL. We also analyzed the properties of the archwires before their usage in the mouth (T0), after one month in the oral cavity (T1), two months (T2) and three months (T3).

The results of the static and dynamic friction coefficients, as well as the friction forces associated with movement, are shown in Table 8. The results reveal statistically significant differences between the CL and SL brackets, with the SL brackets exhibiting the lowest friction and friction forces, and the differences being statistically significant at an alpha level of less than 0.05.

## 4. Discussion

### 4.1. Study Objective and Methodological Considerations

The aim of the present study was to evaluate changes in the superelastic behavior of NiTi orthodontic archwires when used with two different bracket systems—conventional ligation (CL) and self-ligating (SL)—after 1, 2, and 3 months of intraoral service. Unlike most previously published investigations, the archwires analyzed in this study were not subjected to simulated laboratory aging procedures [7,13,16,17,18,19,20,31,32,33,34,35,36,37,38,39]. Instead, the wires were clinically used during orthodontic treatment for defined time intervals before being retrieved and subsequently evaluated under laboratory conditions.

A large proportion of the existing literature has focused on the mechanical behavior of different NiTi phases under controlled laboratory conditions. Consequently, relatively few studies have investigated the mechanical changes that occur in archwires after prolonged exposure to the oral environment, particularly when comparing conventional and self-ligating orthodontic systems. One possible explanation for this limitation is the inherent difficulty of accurately reproducing the complex intraoral environment in experimental setups [21].

With regard to the testing methodology, the evaluation protocol employed in this study was based on the system described by Miura in 1986 [17], which has since become one of the most widely accepted approaches for assessing the bending behavior of orthodontic wires. This method analyzes the relationship between applied load and deflection, allowing a reliable evaluation of the mechanical performance of materials used in orthodontic treatment [16,17,18,40].

Although three-point bending tests remain the most commonly used method to study the behavior of NiTi wires, many laboratory-based studies fail to reproduce the clinical conditions present when an archwire is engaged in a fixed orthodontic appliance system [1,7,12,41]. Consequently, in vivo studies such as the present investigation may provide additional insight into the mechanical alterations that occur during clinical use.

The reason why CL brackets with NiTi archwires produce faster tooth movement than SL brackets is due to the greater friction between the orthodontic wire and the bracket. Because CL brackets create more friction, there is greater force transfer between the wire and the tooth. NiTi wires with SL brackets, having less friction, result in lower load transfer; therefore, the movement is less active, but the superelastic archwire tends to return to its original position, correcting the dental position in the same way as wires with CL brackets [42,43,44,45]. Elastomeric ligatures, as used in conventional ligation systems, generate greater contact area with the archwire and exhibit higher coefficients of friction compared to the clip mechanisms of self-ligating brackets, resulting in significantly higher frictional forces during sliding mechanics [46,47].

### 4.2. Study Limitations

The high interindividual variability of the intraoral environment—including plaque accumulation, salivary pH, oral temperature, and occlusal contact forces—could be considered a limitation of this study, as it hinders the standardization of clinical conditions and may have influenced the mechanical results. Additionally, the two bracket systems evaluated were sourced from different manufacturers, which may introduce a degree of confounding related to bracket design, slot tolerances, and surface characteristics beyond the effect of ligation mode itself. However, both brackets were considered comparable in several key aspects: both feature a 0.022-inch slot, both are regarded as high-quality, premium-range products, and both are manufactured using a similar Metal Injection Molding (MIM) process followed by sintering. While these similarities support their inclusion within the same comparative framework, manufacturer-related differences cannot be entirely ruled out as a potential source of variability. Each retrieved archwire was divided into its right and left halves, which were subsequently tested as separate specimens. Although both halves were considered comparable on the basis of the bilateral symmetry of the study population, strict statistical independence between the two halves of the same archwire cannot be fully guaranteed.

### 4.3. Interpretation of Results and Comparison with Previous Studies

In the present investigation, the mechanical testing protocol followed the recommendations established by ISO 15841:2014 + Amd. 1:2020 [28]. The use of standardized testing conditions is essential to ensure reliable comparisons between different orthodontic wires and experimental groups.

Nine parameters were evaluated in order to determine the slopes of the superelastic plateau and thus characterize the superelastic behavior of the archwires. After one month of intraoral use, statistically significant differences were observed between the CL and SL groups in the mean values of the measured variables (*p* < 0.001). In general terms, the mechanical properties of the archwires in both systems showed significant variations over time.

In the CL group, significant changes were detected between T0 and the first month of intraoral use; however, no further significant changes were observed between the first and second months, suggesting a stabilization of the mechanical properties during this intermediate period. In contrast, the SL group showed a statistically significant change between the baseline measurement (T0) and the first month of intraoral use (T1), after which the mechanical properties remained stable with no further significant differences detected between T1, T2, and T3. After three months of clinical use, the mechanical behavior of the archwires in both groups tended to converge.

When analyzing the plateau slope values, no significant differences were found at deflections of 0.5 mm and 1 mm. However, when a deflection of 2 mm was applied, significantly higher superelastic behavior was observed. This effect was more pronounced in the SL group than in the CL group. When the duration of intraoral exposure was considered, the CL system demonstrated progressive and statistically significant changes in superelastic behavior as time in the oral cavity increased. Conversely, in the SL system, the most notable change occurred after the first month of clinical use, with no additional statistically significant modifications observed thereafter.

Overall, these findings reveal distinct patterns of mechanical degradation depending on the bracket system used. In the CL group, flexural forces and superelastic properties declined progressively and consistently throughout the entire follow-up period, suggesting a cumulative and sustained degradation process with increasing intraoral exposure time. In contrast, the SL group exhibited a marked and rapid reduction in these properties within the first month of intraoral use, after which values stabilized, and no further significant changes were observed. These contrasting trajectories indicate that ligation mode plays a meaningful role in the degradation of the mechanical properties of NiTi archwires under clinical conditions, with CL brackets associated with gradual long-term deterioration and SL brackets with an early but self-limiting mechanical change.

A considerable number of studies have investigated the mechanical behavior and phase transformation characteristics of NiTi wires under in vitro conditions. Despite the extensive evidence generated by orthodontic materials research, laboratory simulations often fail to replicate the complex physical, chemical, and mechanical factors present in the oral environment [38]. The limited number of studies evaluating archwires aged under clinical conditions have mainly focused on corrosion resistance and surface morphology. These surface alterations may influence orthodontic biomechanics by affecting parameters such as friction, superelasticity, and fracture resistance; however, the available clinical evidence remains limited [21,36,37,40,48,49].

It is well established that the mechanical behavior of NiTi wires is influenced by several manufacturing and material-related factors, including chemical composition, thermomechanical treatment, and the degree of cold working during production [40,41]. Other studies have also investigated changes in the superelastic properties of NiTi archwires following sterilization procedures or compared the mechanical behavior of different commercial brands. Some authors have reported statistically significant differences (*p* < 0.001) in the forces generated by various archwire brands during three months of activation and after sterilization procedures. Interestingly, for smaller deflections (<2 mm), many brands were able to recover their mechanical performance after sterilization [50].

In orthodontics, a wide variety of multibracket systems and biomechanical approaches are currently available. Nevertheless, relatively few studies have examined the influence of bracket type on the elastic behavior of NiTi archwires, and most of these investigations have been conducted under in vitro conditions without considering the effects of intraoral aging [19].

Several authors have reported that bracket type can significantly influence the loading and unloading forces generated by archwires. Studies comparing CL and SL systems have demonstrated differences in loading and unloading forces for wires with dimensions of 0.016 in and 0.018 × 0.025 in, with SL brackets generally producing lower loading and unloading forces [36,38,51]. These findings have been attributed mainly to differences in frictional resistance between the archwire, bracket, and ligation system.

In conventional bracket systems, the presence of elastomeric or metallic ligatures can increase friction and may contribute to greater surface wear of the archwire. This increased frictional resistance can modify the mechanical behavior of the wire during both loading and unloading phases. During loading, friction may increase the measured force values, whereas during unloading, it may reduce the effective force delivered by the archwire. In contrast, SL systems typically reduce frictional resistance by eliminating elastomeric ligatures, which could theoretically lead to lower loading forces and higher unloading forces [36,38,52].

However, some studies have reported reductions in both loading and unloading forces when SL brackets are used, suggesting that additional factors may influence the mechanical response of the wire. One possible explanation is the effect of the ligation method on the interbracket span, which determines the effective length of the wire subjected to deflection and therefore influences the magnitude of the generated forces [36,52,53].

Overall, the literature suggests that the mechanical performance of NiTi archwires is influenced by multiple interacting variables, including bracket type, ligation method, intraoral conditions, and duration of clinical use. Nevertheless, further research is required, as relatively few studies have investigated these factors under true clinical conditions. Most available studies have been conducted in vitro and therefore do not fully reproduce the biological and mechanical environment present during orthodontic treatment [7,14,15,16,17,18,19,20,31,32,33,34,35,36,37,38,39].

The results of the present study allow us to reject the null hypothesis, as statistically significant differences in the superelastic properties of NiTi archwires were observed between the self-ligating and conventional bracket groups from the first month of clinical use, although these differences diminished at the third month.

### 4.4. Clinical Implications

The results of the present study indicate that the mechanical properties of NiTi archwires decrease after intraoral use, regardless of the bracket system employed. However, the pattern of change differed between the two techniques. In the CL system, mechanical properties showed a gradual reduction as the duration of intraoral exposure increased. In contrast, the SL system exhibited a more pronounced decrease in properties during the first month of clinical use, followed by relatively stable behavior during the subsequent months.

According to manufacturers’ recommendations, NiTi archwires should remain active for approximately three months to achieve optimal clinical performance [50,51,52,53,54]. If an archwire needs to be removed before this period, CL systems may provide a more favorable option due to their more gradual reduction in superelastic properties over time [55,56]. In comparison, SL systems appear to experience a larger initial decrease in mechanical performance shortly after insertion.

From a clinical perspective, it is also important to consider that archwires previously used in the oral cavity for prolonged periods should not be reused in other patients. The present findings indicate that archwires that have remained intraorally for more than three months show a noticeable reduction in their mechanical and elastic properties, regardless of the bracket system employed.

## 5. Conclusions

The CL group demonstrated a progressive and gradual modification of both flexion and superelastic properties over the three-month (T3) observation period, suggesting a continuous mechanical adaptation of the archwire to the intraoral environment. In contrast, the SL group exhibited the most pronounced changes within the first month (T1) of use, after which the mechanical properties remained relatively stable through the three-month follow-up. This increase in the correction achieved by CL orthodontic systems is due to higher friction coefficients and forces, which transfer a greater load to the teeth than SL bracket systems. However, after three months of use, no differences were found in both techniques.

## Figures and Tables

**Table 1:** Sample distribution by group and time point.

	Time			
	T0	T1	T2	T3
CL Group (30 patients)	10 wires ↓ 20 specimens (*n* = 20)	10 patients ↓ 10 wires ↓ 20 specimens (*n* = 20)	10 patients ↓ 10 wires ↓ 20 specimens (*n* = 20)	10 patients ↓ 10 wires ↓ 20 specimens (*n* = 20)
SL Group (30 patients)		10 patients ↓ 10 wires ↓ 20 specimens (*n* = 20)	10 patients ↓ 10 wires ↓ 20 specimens (*n* = 20)	10 patients ↓ 10 wires ↓ 20 specimens (*n* = 20)

**Table 2:** Results of the force delivered (N) by the orthodontic archwires in the CL group.

CL	Time				Homogeneous Groups Anova < 0.001		
	T0 (*n* = 20)	T1 (*n* = 20)	T2 (*n* = 20)	T3 (*n* = 20)			
Fdef 0.5 mm (N) Mean (SD)	0.089 (0.035)	−0.032 (0.0279)	−0.012 (0.035)	0.0025 (0.030)	T0	T1 T2	T2 T3
Fdef 1 mm (N) Mean (SD)	0.116 (0.034)	0.009 (0.028)	0.0239 (0.036)	0.039 (0.029)	T0	T1 T2	T2 T3
Fdef 2 mm (N) Mean (SD)	0.261 (0.036)	0.162 (0.025)	0.167 (0.041)	0.202 (0.031)	T0	T1 T2	T3
Fdef 3 mm (N) Mean (SD)	0.516 (0.028)	0.478 (0.036)	0.484 (0.037)	0.493 (0.033)	T0 T3	T1 T2 T3	

**Table 3:** Results of the *plateau* slope by the orthodontic archwires in the CL group.

CL	Time				Homogeneous Groups Anova < 0.001			
	T0 (*n* = 20)	T1 (*n* = 20)	T2 (*n* = 20)	T3 (*n* = 20)				
Plateau Slope 0.5 mm Mean (SD)	−0.172 (0.064)	−0.140 (0.068)	−0.262 (0.077)	−0.393 (0.108)	T0 T1	T2	T3	
Plateau Slope 1 mm Mean (SD)	−0.052 (0.030)	−0.145 (0.073)	−0.247 (0.086)	−0.348 (0.094)	T0	T1	T2	T3
Plateau Slope 2 mm Mean (SD)	0.125 (0.066)	−0.015 (0.073)	−0.115 (0.093)	−0.181 (0.099)	T0	T1	T2 T3	

**Table 4:** Results of the force delivered (N) by the orthodontic archwires in the SL group.

SL	Time				Homogeneous Groups Anova < 0.001	
	T0 (*n* = 20)	T1 (*n* = 20)	T2 (*n* = 20)	T3 (*n* = 20)		
Fdef 0.5 mm (N) Mean (SD)	0.089 (0.036)	0.011 (0.044)	−0.012 (0.036)	−0.019 (0.025)	T0	T1 T2 T3
Fdef 1 mm (N) Mean (SD)	0.116 (0.034)	0.045 (0.046)	0.028 (0.043)	0.018 (0.024)	T0	T1 T2 T3
Fdef 2 mm (N) Mean (SD)	0.261 (0.036)	0.212 (0.044)	0.181 (0.045)	0.187 (0.039)	T0	T1 T2 T3
Fdef 3 mm (N) Mean (SD)	0.516 (0.028)	0.486 (0.039)	0.496 (0.033)	0.487 (0.026)	T0 T2	T1 T2 T3

**Table 5:** Results of the plateau slope by the orthodontic archwires in the SL group.

SL	Time				Homogeneous Groups Anova < 0.001	
	T0 (*n* = 20)	T1 (*n* = 20)	T2 (*n* = 20)	T3 (*n* = 20)		
Plateau Slope 0.5 mm Mean (SD)	−0.172 (0.064)	−0.408 (0.099)	−0.385 (0.078)	−0.334 (0.112)	T0	T1 T2 T3
Plateau Slope 1) mm Mean (SD)	−0.052 (0.030)	−0.359 (0.089)	−0.358 (0.079)	−0.317 (0.104)	T0	T1 T2 T3
Plateau Slope 2 mm Mean (SD)	0.125 (0.066)	−0.186 (0.098)	−0.211 (0.088)	−0.158 (0.096)	T0	T1 T2 T3

**Table 6:** Comparison of the force delivered between both appliances (CL and SL). * T-Student 95%. Bonferroni correction α = 0.0023.

		Time			
		T0	T1	T2	T3
Fdef 0.5 mm (N) Mean (SD)	CL	0.089 (0.036)	−0.032 (0.0279)	−0.012 (0.035)	0.0025 (0.030)
	SL		0.011 (0.044)	−0.012 (0.036)	−0.019 (0.025)
	*p*-value *	---	<0.001	0.996	0.020
Fdef 1 mm (N) Mean (SD)	CL	0.116 (0.034)	0.009 (0.028)	0.0239 (0.036)	0.039 (0.029)
	SL		0.045 (0.046)	0.028 (0.043)	0.018 (0.024)
	*p*-value *	---	0.005	0.769	0.017
Fdef 2 mm (N) Mean (SD)	CL	0.261 (0.036)	0.162 (0.025)	0.167 (0.041)	0.202 (0.031)
	SL		0.212 (0.044)	0.181 (0.045)	0.187 (0.039)
	*p*-value *	---	<0.001	0.320	0.171
Fdef 3 mm (N) Mean (SD)	CL	0.516 (0.036)	0.478 (0.036)	0.484 (0.037)	0.493 (0.033)
	SL		0.486 (0.039)	0.496 (0.033)	0.487 (0.026)
	*p*-value *	---	0.492	0.228	0.573

**Table 7:** Comparison of the plateau slope between the two appliances (CL and SL). * T-Student 95%. Bonferroni correction α = 0.0023.

		Time			
		T0	T1	T2	T3
Plateau Slope 0.5 mm	CL	−0.172 (0.064)	−0.140 (0.068)	−0.262 (0.077)	−0.393 (0.108)
	SL		−0.408 (0.099)	−0.385 (0.078)	−0.334 (0.112)
	*p*-value *	---	<0.001	<0.001	0.097
Plateau Slope 1 mm	CL	−0.052 (0.030)	−0.145 (0.073)	−0.247 (0.086)	−0.348 (0.094)
	SL		−0.359 (0.089)	−0.358 (0.079)	−0.317 (0.104)
	*p*-value *	---	<0.001	<0.001	0.327
Plateau Slope 2 mm	CL	0.125 (0.066)	−0.015 (0.073)	−0.115 (0.093)	−0.181 (0.099)
	SL		−0.186 (0.098)	−0.211 (0.088)	−0.158 (0.096)
	*p*-value *	---	<0.001	0.002	0.470

**Table 8:** Static (m_s_) and dynamic (m_d_) friction coefficients and friction force between the NiTi archwires and the CL and SL brackets. The asterisk indicates a statistically significant difference in the values with *p* < 0.05.

Orthodontic System	m_s_	m_d_	F _friction_ (N)
NiTi-CL	0.27 ± 0.07	0.11 ± 0.07	10.23 ± 2.12
NiTi-SL	0.09 ± 0.04 *	0.08 ± 0.04 *	2.69 ± 0.98 *

## Data Availability

The authors can provide details of the research requirements by letter and comments under needs.

## Abbreviations

The following abbreviations are used in this manuscript:

CL	Conventional ligation brackets
SL	Self-ligating brackets
NiTi	Nickel Titanium
CUO	Dentistry University Clinic
Fdef	Force delivered in Newtons
Sp	Deflection at the end of the Plateau slope
